# Regional Homogeneity Changes in Hemodialysis Patients with End Stage Renal Disease: *In Vivo* Resting-State Functional MRI Study

**DOI:** 10.1371/journal.pone.0087114

**Published:** 2014-02-06

**Authors:** Cheng Li, Huan-Huan Su, Ying-Wei Qiu, Xiao-Fei Lv, Sheng Shen, Wen-Feng Zhan, Jun-Zhang Tian, Gui-Hua Jiang

**Affiliations:** 1 Department of Renal Transplantation, Guangdong No.2 Provincial People's Hospital, Guangzhou, People's Republic of China; 2 Department of Medical Imaging, Guangdong No.2 Provincial People's Hospital, Guangzhou, People's Republic of China; 3 State Key Laboratory of Oncology in South China and Department of Medical Imaging and Interventional Radiology, Sun Yat-Sen University Cancer Center, Guangzhou, People's Republic of China; Institute of Psychology, Chinese Academy of Sciences, China

## Abstract

**Objective:**

To prospectively investigate and detect early cerebral regional homogeneity (ReHo) changes in neurologically asymptomatic patients with end stage renal disease (ESRD) using in vivo resting-state functional MR imaging (Rs-fMRI).

**Methods:**

We enrolled 20 patients (15 men, 5 women; meanage, 37.1 years; range, 19–49 years) with ESRD and 20 healthy controls (15 men, 5 women; mean age, 38.3 years; range, 28–49 years). The mean duration of hemodialysis for the patient group was 10.7±6.4 monthes. There was no significant sex or age difference between the ESRD and control groups. Rs-fMRI was performed using a gradient-echo echo-planar imaging sequence. ReHo was calculated using software (DPARSF). Voxel-based analysis of the ReHo maps between ESRD and control groups was performed with a two-samples *t* test. Statistical maps were set at *P* value less than 0.05 and were corrected for multiple comparisons. The Mini-Mental State Examination (MMSE) was administered to all participants at imaging.

**Results:**

ReHo values were increased in the bilateral superior temporal gyrus and left medial frontal gyrus in the ERSD group compared with controls, but a significantly decreased ReHo value was found in the right middle temporal gyrus. There was no significant correlation between ReHo values and the duration of hemodialysis in the ESRD group. Both the patients and control subjects had normal MMSE scores (≥28).

**Conclusions:**

Our finding revealed that abnormal brain activity was distributed mainly in the memory and cognition related cotices in patients with ESRD. The abnormal spontaneous neuronal activity in those areas provide information on the neural mechanisms underlying cognitive impairment in patients with ESRD, and demonstrate that Rs-fMRI with ReHo analysis is a useful non-invasive imaging tool for the detection of early cerebral ReHo changes in hemodialysis patients with ESRD.

## Introduction

End stage renal disease (ESRD) is defined as a glomerular filtration rate (GFR) less than 15 mL/min/1.73 m^2^, or chronic renal failure that has progressed to the point at which the kidneys are permanently functioning at less than 10% of their capacity. Patients with ESRD usually have central nervous system abnormalities, some related to ESRD itself and others related to problems secondary to hemodialysis [1], [2], [3], [4]. These Patients with ESRD often present with neurological complications such as focal white matter lesions, cerebral atrophy, osmotic demyelination syndrome, dialysis encephalopathy, hypertensive encephalopathy, intracranial hemorrhage, infarction, sinus thrombosis, and infection [1], [2], [3], [4]. Furthermore, among patients with ESRD, depression and cognitive impairment are the most common causes of neuropsychiatric illness [5]. Cognitive deficits may occur in patients with ESRD long before any overt neurological symptoms are observed. Depression and cognitive impairment are considered important factors for the determination of a patient's survival and prognosis. Thus, it is important to elucidate the mechanism of depression and cognitive impairment in Patients with ESRD.

Previous studies of brain changes that accompany or follow ESRD have mainly used brain computed tomography (CT) or conventional magnetic resonance imaging (MRI) [6], [7], [8], [9], [10]. Computed tomography can be used to assess neurological complications, such as intracranial hemorrhage and cerebral infarction. MRI is a sensitive imaging tool for the neurological evaluation of patients with ESRD [11]. Because the clinical evaluation and ongoing assessment of ESRD are complicated, so it has been suggested that MRI be used before the onset of therapy, so that these initial findings can serve as a basis for later comparisons [3], [4], [11]. For example, conventional MRI studies have shown focal white matter lesions to be more common in patients undergoing hemodialysis (56%) than in the normal population (27%) [11]. In the past decade, functional imaging studies about ESRD have consistently demonstrated regional microscopic structure and metabolic abnormalities in the white matter. Hsieh et al, who used diffusion tensor imaging to measure fractional anisotropy (FA) values in patients with ESRD, reported that patients with ESRD have significant lower FA values than healthy control subjects [12]. Chiu et al reported that significant elevations of the choline/phosphatidylcholine (Cho)/total cholesterol (tCr) and myo-inositol (mI)/tCr ratios in the frontal grey matter, frontal white matter, and temporal white matter as well as in the basal ganglia were found in ESRD group compared with controls [13]. However, these brain imaging methods have limitation in that they are not capable of estimation and visualization of neural activity.

Functional brain imaging studies have suggested that the brain is not inactive during rest, but rather shows a default state of activation [14], [15], [16], [17], [18]. Low frequency oscillations (ranging from 0.01 to 0.1 Hz) of resting-state functional MRI (Rs-fMRI) time-series are known to show correlated patterns between anatomically separated brain regions [18], [19], [20]. It has been suggested that these correlations originate from coherency in the underlying neuronal activation patterns of these regions and reflect functional connectivity. Regions that show this kind of coherent functional behavior are said to form a resting-state network (RSN). Regional homogeneity (ReHo) measures the functional coherence of a given voxel with its nearest neighbors and can be used to evaluate resting-state brain activities based on the hypothesis that significant brain activities would more likely occur in clusters than in a single voxel [21]. The Kendall coeffi cient of concordance was used to measure the similarity of the time series of one voxel with that of its nearest neighbors in a voxel-wise analysis [22]. Regional homogeneity does not require the onset time of stimulus and therefore is useful for Rs-fMRI data analysis. Regional homogeneity has been successfully used to study the functional modulations in the resting state in patients with Alzheimer disease [23], Parkinson disease [24], schizophrenia [25], and neuromyelitis optica [26] and in healthy aging subjects [27]. Regional homogeneity could be regarded as a measure for investigating human brain activities in the resting state. It may be helpful to understand the pathophysiology of cognitive deficits in patients with ESRD using the ReHo method, which can reflect the temporal homogeneity of neural activity. In this prospective study, we characterized and compared ReHo differences between hemodialysis patients with ESRD and healthy control subjects using Rs-fMRI to understand the effect of ESRD on brain function.

## Materials and Methods

### Subjects

This prospective study was approved by the Research Ethics Review Board of the Institute of Mental Health at the Guangdong No. 2 Provincial People's Hospital. Written informed consent was obtained from all subjects. For this hospitalbased prospective case-control study, we recruited 23 patients with ESRD from the renal transplantation department at our hospital and 20 healthy volunteers with normal renal function between August 2011 and July 2012. To avoid possible confounding effects, all participants were younger than 50 years. They were excluded if they had a history of diabetes, alcoholism, drug abuse, psychiatric disorders, or major neurologic disorders (severe head injury, stroke, epilepsy, or visible lesions). Conventional MR images were interpreted by an experienced radiologist (J.Z.T) with 20 years of experience in neuroradiology who was blinded to whether the images were from the patient group or the control group. Subjects with brain lesions at conventional T1 or T2-fluid-attenuated inversion recovery (FLAIR) MR imaging were excluded. Three patients, whose T2-FLAIR MR images showed abnormal hyperintensities, were excluded because the imaging evidence suggested infarcts, one of the exclusion criteria. The final study population included 20 patients with ESRD (15 men, 5 women) and 20 healthy controls (15 men, 5 women). All patients were diagnosed with renal failure by GFR less than 15 mL/min/1.73 m^2^, and underwent regular hemodialysis. Each subject completed a questionnaire before MRI examination, including age, sex, years of education, duration of hemodialysis and Mini-Mental State Examination (MMSE).

### Imaging studies

MRI data were obtained using a 1.5T MR scanner (Achieva Nova-Dual; Philips, Best, the Netherlands) in the Department of Medical Imaging, Guangdong No. 2 Provincial People's Hospital. Each subject lay supine with the head comfortably fixed using a belt and foam pads. During Rs-fMRI, subjects were instructed to close their eyes and remain as quiet as possible and to not think of anything systematically or fall asleep. The conventional imaging sequences including T1-weighted images and T2-FLAIR images were obtained for every subject to detect clinically silent lesions. Rs-fMRI data were acquired using a gradient-echo echo-planar sequence sensitive to blood oxygenation level dependent (BOLD) contrast. The Rs-fMRI acquisition parameters were as follows: repetition time (TR)  = 3,000 ms, echo time (TE)  = 50 ms, flip angle  = 90°, field-of-view  = 230×230 mm^2^, matrix  = 64×64, and total volumes  = 160. A total of 33 axial slices of 4.5 mm thickness were collected with no intersection gap. In-plane resolution was 3.59×3.59 mm^2^. Each Rs-fMRI scan lasted 8 minutes. After the examination, all participants were asked questions to verify the degree of their cooperation.

### Data preprocessing and regional homogeneity calculation

The imaging data were preprocessed mainly using a MATLAB toolbox called Data Processing Assistant for Resting-State (DPARSF [28]; http://restfmri.net/forum/DPARSF) for “pipeline” data analysis of Rs-fMRI. DPARSF is based on statistical parametric mapping software functions (SPM8; http://www.fil.ion.ucl.ac.uk/spm) and REST software [29]; http://resting-fmri.sourceforge.net). For each participant, the first 10 time points were discarded to avoid transient signal changes before magnetization reached steady-state and to allow subjects to become accustomed to the fMRI scanning noise. The raw data were corrected for acquisition delay between slices and for the head motion (a least squares approach and a 6 parameter spatial transformation). Subjects with head motion exceeding 1.5 mm in any dimension through the resting-state run were removed. Following the motion correction, all data were spatial normalized to the Montreal Neurological Institute (MNI) template (resampling voxel size  = 3×3×3 mm^3^). Subsequent data preprocessing included removal of linear trends and temporal filtering (band pass, 0.01 to 0.08 Hz) to remove the effects of very low-frequency drift and physiological high frequency respiratory and cardiac noise for further ReHo analysis [18].

The ReHo calculation procedure was the same as that reported in a previous study [21]. Briefly, this is accomplished on a voxel-by-voxel basis by calculating Kendall's coefficient of concordance (KCC) [22] for a given voxel time series with those of its nearest 26 neighbors.
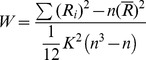



where *W* is the KCC among given voxels, ranging from 0 to 1; R_i_ is the sum rank of the with time point (R_i_ = 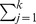
r*_ij_* where r*_ij_* is the rank of the *i*th time point in the *j*th voxel); 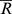 =  ((n+1) *k*)/2 is the mean of the R_i_; *k* is the number of time series within a measured cluster (27, one given voxel plus the number of its neighbors); and *n* is the number of ranks (n = 150 for this study). The KCC value was calculated to this voxel, and an individual KCC map was obtained for each subject. To reduce the influence of individual variations in the KCC value, ReHo maps normalization was preformed by dividing the KCC among each voxel by the averaged KCC of the whole brain. The resulting data were then spatially smoothed with an 8-mm full-width at half-maximum (FWHM) Gaussian kernel to reduce noise and residual differences in gyral anatomy.

### Statistical analysis

Two-sample *t*-tests were performed to assess the differences in age, duration of education and number of cigarettes smoked per day between Patients with ESRD and healthy subjects. Mann-Whitney U tests were used to analyze the differences in sex between the two groups. Pearson correlation coefficient was used to analyze the association between ReHo values and the duration of hemodialysis in the patient group. Analyses were conducted using software (SPSS, version 13.0; Chicago, Ill, USA), and a *P* value less than 0.05 (two-tailed) was considered statistically significant.

A second-level random-effect one-sample *t*-test (*P*<0.01, with False Discovery Rate (FDR) correction) was performed to show the ReHo results with in each group. To explore the ReHo differences between Patients with ESRD and healthy subjects, a second-level random-effect two-sample *t*-test was performed on the individual normalized ReHo maps in a voxel-by-voxel manner by taking age and years of education as confounding covariates. Significant differences were set at the threshold of a corrected cluster level at *P* less than 0.05. Threshold correction was performed by using a program (AlphaSim; Analysis of Functional NeuroImages, http://afni.nimh.nih.gov/afni/) that applies Monte Carlo simulation to calculate the probability of false positive detection by taking into consideration both the individual voxel probability threshold and cluster size [30]. Using this program, a corrected significance level of *P* less than 0.05 was obtained by clusters with a minimum volume of 1998 mm^3^ at an uncorrected individual voxel height threshold of *P*<0.01. This enabled the identification of significant changes in ReHo in the Patients with ESRD compared with the controls. The parameters were as follow: individual voxel *P* value = 0.01, 1,000 simulations, FWHM = 8 mm, and whole brain mask.

## Results

**Figure 1:**
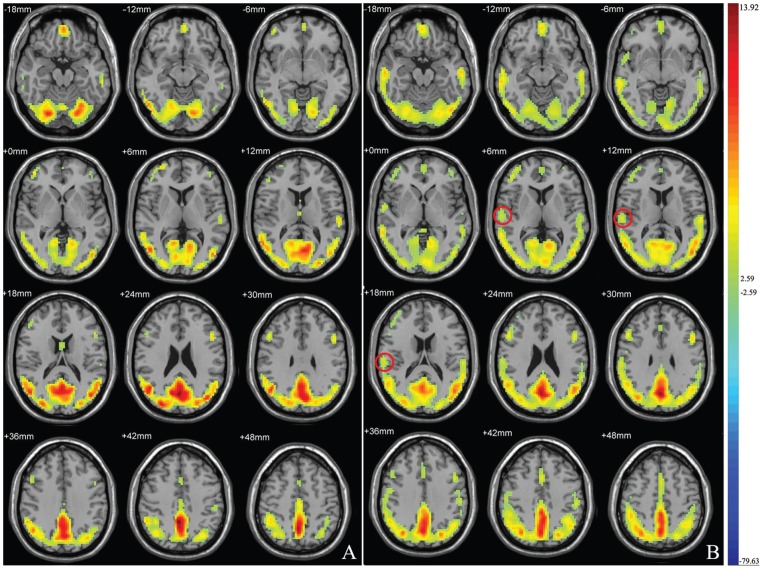
Mean ReHo maps within the ESRD group (A) and healthy controls (B). Left side of the images corresponds to the right side of the subjects. T-score bars are shown on the right. The images illustrate high ReHo in the default network. In the controls group but not the patient group, high ReHo was shown in the right middle temporal gyrus.

**Figure 2:**
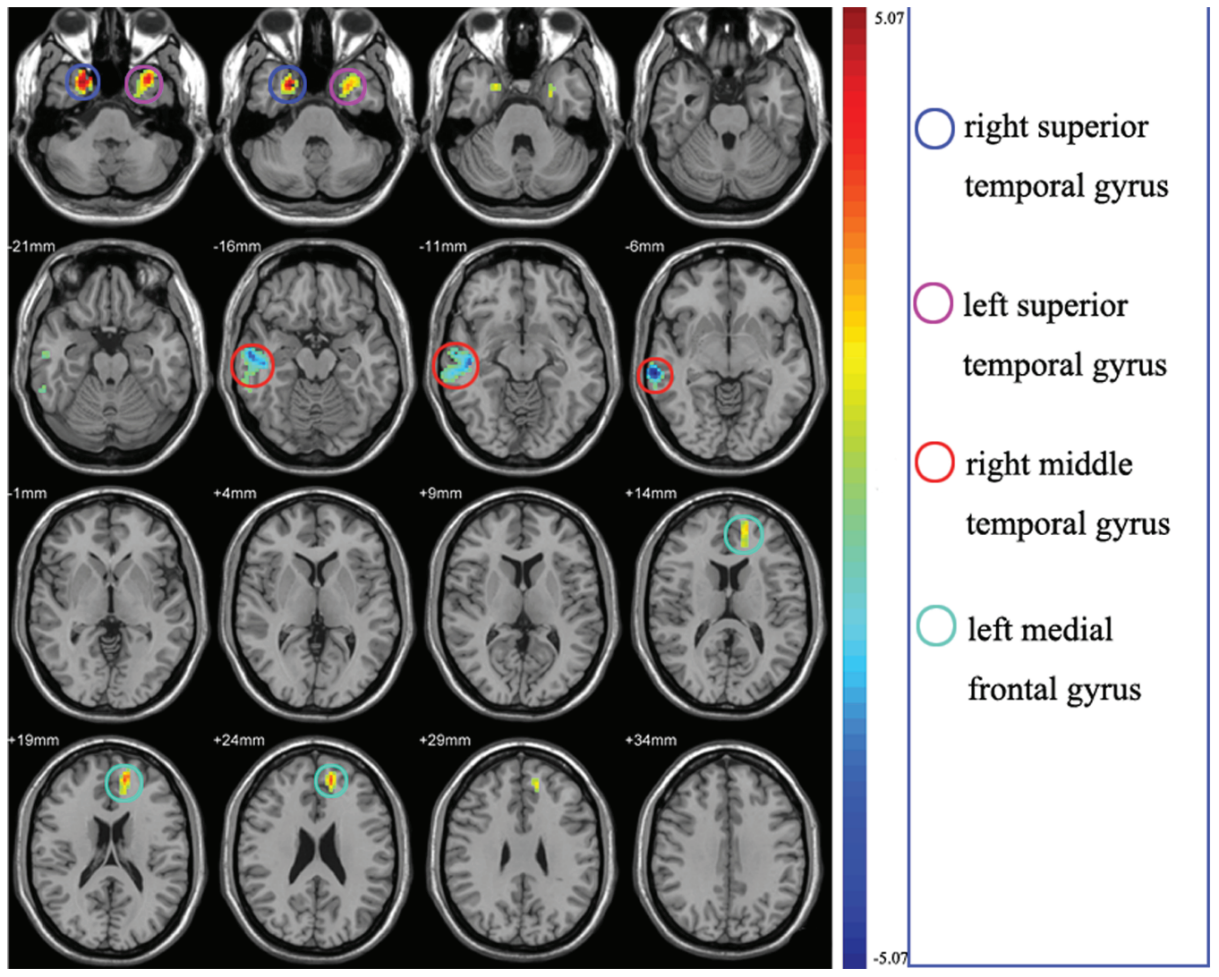
Maps showing statistically significant differences between the ESRD group and the control group. The Patients with ESRD showed a significant ReHo increase in the bilateral superior temporal gyrus and left medial frontal gyrus (warm colors), but a decrease in the right middle temporal gyrus (cold colors) (n = 20, corrected *P*<0.05).

After exclusion of three patients with hyperintensities on T2-FLAIR images, we found no other abnormality in morphology or signal intensity on T1-weighted and T2- FLAIR images. The mean age of the patients and healthy controls was 37.1±8.6 years (range, 19–49 years) and 38.3±6.5 years (range, 28–49 years), respectively. Three subjects in the ESRD group were outpatients, we could not collect the duration of hemodialysis of them. The mean duration of hemodialysis for the patient group (n = 17) was 10.7±6.4 monthes. There were no significant differences in age, sex, years of education and number of cigarettes smoked per day between patients with ESRD and control subjects (Table. 1). The mean ReHo maps with in each group are shown in Figure. 1, illustrating high ReHo in the default network. In the controls group, high ReHo in the right middle temporal gyrus was shown, but this was not found in the patient group. Compared with the control group, the Patients with ESRD showed a significant ReHo increase in the bilateral superior temporal gyrus (*P*<0.05, AlphaSim corrected) and left medial frontal gyrus (*P*<0.05, AlphaSim corrected), but a decrease in the right middle temporal gyrus (*P*<0.05, AlphaSim corrected) (Table. 2, Figure. 2). There was no significant correlations between ReHo values and the duration of hemodialysis in the ESRD group. No significantly different score was found between patient and control groups, all subjects had normal MMSE scores (≥28).

**Table 1:** Demographic and clinical characteristics of ESRD and control groups.

Characteristic	ESRD group(n = 20)	Control group(n = 20)	*t* value	*P* value*
Age(y)	37.1±8.6	38.3±6.5	−0.499	0.621
Sex#				1.000
male	15	15		
female	5	5		
Education(y)	12.0±2.9	12.9±3.2	−0.938	0.354
No. of cigarettes/day▴	13.5±9.2	16.7±12.6	−0.325	0.768
Duration of dialysis(mo)$	10.7±6.4	NA		
MMSE score	29.2±0.8	29.5±0.8	−1.189	0.242

Unless otherwise indicated, data are mean±standard deviations.

NA = not applicable. MMSE  =  Mini-mental status examination.

^*^ *P* values are two sided.

# For sex composition, χ^2^ = 0.000 and ν = 1.

▴ There were two patients in the ESRD group and three persons in the control group with a history of smoking.

$ There were 17 inpatients in the ESRD group.

**Table 2:** Brain regions with abnormal ReHo in patients with ESRD compared with control subjects.

			Mean Reho value			MNI coordinate			
Brain area	No. of voxels	Side	Control group	ESRD group	Brodmann area	x	y	z	Peak t value
Superior Temporal gyrus	35	R	−0.440±0.062	−0.329±0.076	38	24	9	−39	5.071
Superior Temporal gyrus	42	L	−0.366±0.074	−0.243±0.101	38	−33	12	−42	4.594
Middle Temporal gyrus	132	R	0.099±0.049	−0.033±0.072	21	66	−30	−6	−5.018
Medial Frontal gyrus	53	L	−0.239±0.058	−0.145±0.082	9	−12	48	21	4.052

L = left, R = right, MNI = Montreal Neurological Institute.

## Discussion

The ReHo is a data-driven method, which assumes that a given voxel is temporally similar to its neighbors. It measures the ReHo of the time series of the regional BOLD signal. Therefore, ReHo reflects the temporal homogeneity of the regional BOLD signal rather than its density. As the BOLD signal of fMRI may reflect neural activity [31], abnormal ReHo is possibly relevant to the changes of temporal aspects of neural activity in the regional brain. Therefore ReHo may detect the brain regions with abnormal activity.

We found ReHo values in patients with ESRD to be significantly increased in the bilateral superior temporal gyrus and left medial frontal gyrus, but decreased in the right middle temporal gyrus. Interestingly, all of the brain areas with significant ReHo changes were located in the temporal and frontal lobes, which are closely related to the memory and cognition. Our findings are similar to those of many previous brain imaging studies in pre-dialysis patients. Using Tc-99m ethylcysteinate dimer brain single photon emission tomography, Song et al found that prior to beginning dialysis patients with chronic kidney disease have significant hypoperfusion in the right superior and middle temporal gyrus and inferior frontal gyrus [32]. In their previous study using F-18-fluorodeoxyglucose positron emission tomography, they found that several voxel clusters had significantly decreased cerebral glucose metabolism in patients with chronic kidney disease who had not started dialysis, including the prefrontal cortex, superior temporal gyrus and middle temporal gyrus [33]. Kim et al reported that depressive mood and anxiety factors were negatively correlated with regional cerebral blood flow in bilateral superior temporal gyrus, right middle temporal gyrus and left superior frontal gyrus [34]. Thus, all of them cannot reflect the baseline directly as resting-state studies can.

Recently, Xue Liang et al did a similar research to us. They used Rs-fMRI with ReHo algorithm to investigate the pattern of spontaneous neural activity in patients with ESRD. In their study, they found both MNE (minimal nephro-encephalopathy) and non-NE (non-nephro-encephalopathy) patients show decreased ReHo in the multiple areas of bilateral frontal, parietal and temporal lobes [35]. These results have a few difference to ours. It may be relate to the small sample size. Whatever, the differences in ReHo indicate a poor level of coordination and a disorder of communication among neurons in the brain region [21]. These changes in ReHo suggest an abnormality in the resting-state brain function of patients with ESRD and may be early signs for the development of uremic encephalopathy or dialysis encephalopathy.

The temporal and frontal regions are considered as important components of human default-mode networks [15], [17], [36], and have been shown to exhibit mild cognitive impairment (MCI)-related structural and functional abnormalities [37], [38], [39], [40], [41]. James et al reported that hemodialysis patients have a high prevalence of mild cognitive impairment (MCI) despite normal global cognitive function [42]. MCI is a transitional state between normal cognition and the earliest clinical features of dementia. MCI is likely under-diagnosed but highly prevalent in dindividuals with ESRD [43], [44], [45], and Murray et al described MCI in nearly 64% of hemodialysis patients [46]. In the present study, we found that patients with ESRD have normal global cognitive function (MMSE score >25), but that does not mean that these patients are free of MCI. The greatest defect in our research was that we did not assess the MCI in patients with ESRD.

In our study, there was no significant correlation between ReHo values and the duration of hemodialysis in the ESRD group. This finding may indicate that the significant change in ReHo values relates to ESRD itself but not hemodialysis.

Some limitations of our study are worth mentioning. First, we did not discriminate between neuronal versus vascular effects on changes in ReHo values in patients with ESRD. Second, the sample size in this study is relatively small, and thus the results of the current study may no be representative of ESRD in general. Third, the lack of MCI examinations in our sample is the most serious drawback of our study. Fourth, we did not collect follow-up Rs-fMRI data after renal replacement. In future studies, examinations should include not only fMRI but also arterial spin labeling perfusion imaging, electroencephalograms and other measures to discriminate between neuronal versus vascular effects on changes in neural activity in patients with ESRD. More attention also needs to be paid to mental test and serial changes in neural activity after renal replacement therapy in Patients with ESRD.

In conclusion, we found significant change in ReHo values in patients with ESRD in brain areas located in temporal and frontal lobes, which are closely related to the memory and cognition. The abnormal spontaneous neuronal activity in those areas may be the neural mechanisms underlying the cognitive impairment in patients with ESRD, and suggested that an abnormal ReHo value in certain brain areas may be a potential biomarker to detect the early cerebral ReHo changes in hemodialysis patients with ESRD.
